# Promoter methylation of *RNF180* is associated with *H.pylori* infection and serves as a marker for gastric cancer and atrophic gastritis

**DOI:** 10.18632/oncotarget.8523

**Published:** 2016-04-01

**Authors:** Fang Han, Li-ping Sun, Shuang Liu, Qian Xu, Qiao-yi Liang, Zhe Zhang, Hai-chao Cao, Jun Yu, Dai-ming Fan, Yong-zhan Nie, Kai-chun Wu, Yuan Yuan

**Affiliations:** ^1^ Tumor Etiology and Screening Department of Cancer Institute and General Surgery, The First Affiliated Hospital of China Medical University, and Key Laboratory of Cancer Etiology and Prevention (China Medical University), Liaoning Provincial Education Department, Shenyang, Liaoning, China; ^2^ Institute of Digestive Disease and Department of Medicine and Therapeutics, State Key Laboratory of Digestive Disease, Li Ka Shing Institute of Health Sciences, CUHK Shenzhen Research Institute, The Chinese University of Hong Kong, Hong Kong; ^3^ State Key Laboratory of Cancer Biology and Xijing Hospital of Digestive Diseases, Xijing Hospital, Fourth Military Medical University, Xi'an, Shanxi, China

**Keywords:** gastric cancer, atrophic gastritis, bisulfite genomic sequencing, CpG islands, methylation

## Abstract

Promoter methylation (PM) of RING-finger protein (*RNF*) *180* affects gastric cancer (GC) prognosis, but its association with risk of GC or atrophic gastritis (AG) is unclear. We investigated relationships between *RNF180* PM and GC or AG, and the effects of *Helicobactor pylori (H.pylori)* infection on *RNF180* PM. This study included 513 subjects (159 with GC, 186 with AG, and 168 healthy controls [CON]) for RNF180 PM analysis, and another 55 GC patients for RNF180 gene expression analysis. Methylation was quantified using average methylation rates (AMR), methylated CpG site counts (MSC) and hypermethylated CpG site counts (HSC). *RNF180* promoter AMR and MSC increased with disease severity. Optimal cut-offs were GC + AG: AMR > 0.153, MSC > 4 or HSC > 1; GC: AMR > 0.316, MSC > 15 and HSC > 6. Hypermethylation at 5 CpG sites differed significantly between GC/AG and CON groups, and was more common in GC patients than AG and CON groups for 2 other CpG sites. The expression of RNF180 mRNA levels in tumor were significantly lower than those in non-tumor, with the same as in hypermethylation than hypomethylation group. *H.pylori* infection increased methylation in normal tissue or mild gastritis, and increased hypermethylation risk at 3 CpG sites in AG. In conclusion, higher AMR, MSC and HSC levels could identify AG + GC or GC. Some *RNF180* promoter CpG sites could identify precancerous or early-stage GC. *H.pylori* affects *RNF180* PM in normal tissue or mild gastritis, and increases hypermethylation in 3 CpG sites in AG.

## INTRODUCTION

Aberrant CpG island methylation, especially in promoter regions of tumor suppressor genes, is related to tumorigenesis. For example, *RUNX3* promoter methylation (PM) is related to esophageal squamous cell carcinoma [1]. *WIF1* and *DKK3* to poor prognosis in breast cancer [2]; *FGR3* mutation and hypermethylation to bladder cancer [3] and *SFRP2* hypermethylation to gastric cancer (GC) [4, 5]. CpG island methylation can inactivate or downregulate tumor suppressor genes, ultimately leading to tumor development and progression. In recent years, relationships between the PM of tumor suppressor genes and carcinoma risk have attracted much attention in methylation research [6, 7].

Really Interesting, new gene (RING) Finger (RNF) is a family of ubiquitin ligases that function as tumor suppressors [8–10]. RNF180 is a recently discovered member of the RNF family—an E3 ubiquitin ligase that reportedly participates in proliferation and differentiation [11]. Although the PM status of *RNF180* is known to affect GC prognosis [12–14]; however, its effect on risk of GC, or on its most important precancerous state, atrophic gastritis (AG), remain unclear.

*Helicobactor pylori (H.pylori)* is a major carcinogen of gastric cancer and can also lead to abnormal DNA methylation [15–19]. Whether *H.pylori* infection would modify *RNF180* DNA methylation has not been revealed yet. This study focused on the relationships between *RNF180* PM and risk of GC and AG, and the effect of *H.pylori* infection on *RNF180* PM.

## RESULTS

### Relationship between AMR of RNF180 promoter and GC or AG

**Figure 1:**
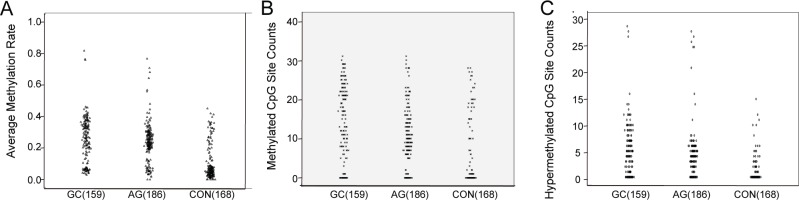
The scatter plot of RNF180 gene promoter methylation (**A**) The average methylation rate of *RNF 180* gene in different gastric diseases; (**B**) The methylated CpG site counts of *RNF 180* gene in different gastric diseases; (**C**) The hypemethylated CpG site counts *RNF 180* gene in different gastric diseases.

We amplified the *RNF180* promoter area from −224 bp to +94 bp (a 318-bp fragment). The results show AMR in the *RNF180* promoter to increase with disease severity, such that CON < AG < GC (GC vs. AG: *P* < 0.021; AG vs. CON: *P* < 0.001; Figure 1A).

**Figure 2:**
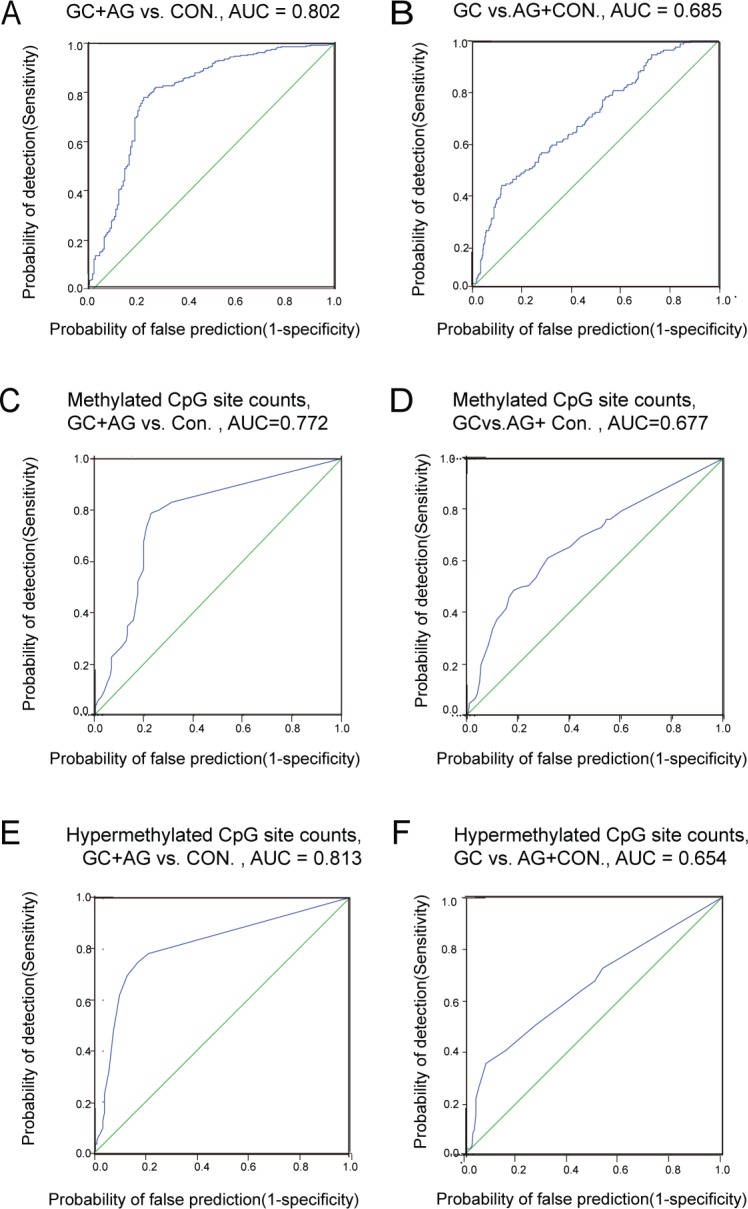
The ROC curve of RNF180 gene promoter methylation (**A**) GC + AG vs. CON., AUC = 0.802; (**B**) GC vs. AG + Con., AUC = 0.685; (**C**) Methylated CpG site counts, GC + AG vs. CON., AUC = 0.772; (**D**) Methylated CpG site counts, GC vs. AG + CON., AUC = 0.677; (**E**) Hypermethylated CpG site counts, GC + AG vs. CON., AUC = 0.813; (**F**) Hypermethylated CpG site counts, GC vs. AG + CON AUC = 0.654.

We also evaluated the validity of *RNF180* promoter area AMR in distinguishing AG or GC from controls, by ROC curves. We found that AMR > 0.153 was 78.6% sensitive and 77.4% specific (AUC = 0.802) in distinguishing [GC + AG] from controls (Figure 2A); and at 0.316, was 41.5% sensitive and 87.9% specific for GC vs. [AG + CON] (Figure 2B).

### Relationships between MSC and HSC of the RNF180 promoter area and GC or AG

Relationships between MSC or HSC and GC or AG were analyzed by the Mann–Whitney *U* test. As with AMR, MSC increased with disease severity (i.e., CON < AG < GC, Figure 1B). However, HSC in the GC and AG groups were higher than for controls, (*P* < 0.001 for both), but did not significantly differ between the GC and AG groups (*P* = 0.301; Figure 1C).

We further evaluated *RNF180* promoter MSC and HSC as biomarkers for AG or GC, using ROC curves. We found MSC > 4 was 78.6% sensitive and 77.4% specific (AUC = 0.772) in distinguishing [GC + AG] groups from controls (Figure 2C); MSC > 15 was 48.4% sensitive and 87.9% specific (AUC = 0.772) in distinguishing GC from [AG + CON] (Figure 2D). We found that HSC > 1 was 74.5% sensitive and 83.3% specific (AUC = 0.813) in distinguishing GC+AG from controls (Figure 2E); and at HSC > 6, was 35.2% sensitive and 92.1% specific(AUC = 0.654) at distinguishing GC from [AG + CON] (Figure 2F).

### Relationship between hypermethylated of RNF180 promoter CpG sites and GC or AG

The location of every hypermethylation site in *RNF180* gene and distribution of hypermethylation frequency in GC, AG and control groups were shown in Supplementary Table S1, Based on a comparative analysis of relationships between MSC or HSC and GC or AG, we selected 8 CpG sites with which hypermethylation frequencies > 25% among a total 513 individuals—M3(−165), M5(−148), M7(−133), M8(−130), M20(−57), M25(−34), M27(−26) and M30(+5)—to evaluate the risk of AG or GC. All results were analyzed using logistic regression. *P* values and odd ratio (OR) were adjusted by sex, age, smoking, drinking and *H.pylori* infection.

We found hypermethylation frequencies of M5(−148) and M27(−26) in the GC group were higher than in the AG + CON group, and were associated with increased GC risks of 5.85 folds (95% confidence interval [CI]: 3.20–10.69) and 4.27 folds (95% CI: 2.54–7.19), respectively. We also found that hypermethylation in 5 CpG sites—M3(−165), M7(−133), M20(−57), M25(−34) and M30(+5)—could increase the risks for AG and GC and there is significant difference when compares to random two groups of GC AG CON(GC vs. CON, AG vs. CON and GC vs. AG, Table 1).

**Table 1:** Association between hypermethylation frequency and risks of gastric cancer and atrophic gastritis

					GC vs CON		GC vs AG		AG vs CON		GC vs AG + CON	
		GC	AG	CON	*P*^a^	OR (95% CI)	*P*	OR (95% CI)	*P*	OR (95% CI)	*P*	OR (95% CI)
M3	L^b^	101 (63.5)	76 (40.9)	160 (95.2)		1 (Ref.)		1 (Ref.)		1 (Ref.)		1 (Ref.)
	H^c^	58 (36.3)	110 (59.1)	8 (4.8)	< 0.001	11.43 (4.95, 26.41)	< 0.001	0.36 (0.22, 0.59)	< 0.001	25.05 (10.51, 59.71)	0.981	0.98 (0.64, 1.51)
M5	L	120 (75.5)	171 (92.4)	163 (97.0)		1 (Ref.)		1 (Ref.)		1 (Ref.)		1 (Ref.)
	H	39 (24.5)	14 (7.6)	5 (3.0)	< 0.001	10.28 (3.70, 28.56)	< 0.001	3.86 (1.94, 7.68)	0.083	2.91 (0.87, 9.70)	< 0.001	5.85 (3.20, 10.69)
M7	L	117 (73.6)	77 (41.6)	160 (95.2)		1 (Ref.)		1 (Ref.)		1 (Ref.)		1 (Ref.)
	H	42 (26.4)	108 (58.4)	8 (4.8)	< 0.001	7.26 (3.12, 16.89)	< 0.001	0.23 (0.14, 0.37)	< 0.001	22.70 (9.93, 51.95)	0.028	0.60 (0.38, 0.95)
M8	L	122 (76.7)	127 (68.6)	160 (95.2)		1 (Ref.)		1 (Ref.)		1 (Ref.)		1 (Ref.)
	H	37 (23.3)	58 (31.4)	8 (4.8)	< 0.001	6.43 (2.74, 15.27)	0.065	0.61 (0.37, 1.03)	< 0.001	9.28 (3.95, 21.84)	0.406	1.23 (0.76, 1.98)
M20	L	111 (69.8)	108 (58.4)	158 (94.0)		1 (Ref.,		1 (Ref.)		1 (Ref.)		1 (Ref.)
	H	48 (30.2)	77 (41.6)	10 (6.0)	< 0.001	5.22 (2.42, 11.27)	0.012	0.54 (0.33, 0.87)	< 0.001	7.78 (3.60, 16.82)	0.608	1.13 (0.72, 1.76)
M25	L	78 (49.1)	53 (28.6)	145 (86.3)		1 (Ref.)		1 (Ref.)		1 (Ref.)		1 (Ref.)
	H	81 (50.9)	132 (71.4)	23 (13.7)	< 0.001	5.82 (3.24, 10.47)	< 0.001	0.411 (0.26, 0.66)	< 0.001	10.77 (5.88, 19.71)	0.326	1.23 (0.82, 1.84)
M27	L	113 (71.1)	165 (89.2)	157 (93.5)		1 (Ref.)		1 (Ref.)		1 (Ref.)		1 (Ref.)
	H	46 (28.9)	20 (10.8)	11 (6.5)	< 0.001	5.29 (2.44, 11.44)	< 0.001	3.16 (1.72, 5.80)	0.067	2.31 (0.94, 5.64)	< 0.001	4.27 (2.54, 7.19)
M30	L	82 (51.6)	48 (25.9)	150 (89.3)		1 (Ref.)		1 (Ref.)		1 (Ref.)		1 (Ref.)
	H	77 (48.4)	137 (74.1)	18 (10.7)	< 0.001	7.13 (3.81, 13.36)	< 0.001	0.31 (0.19, 0.51)	< 0.001	15.28 (8.07, 28.93)	0.68	1.09 (0.73, 1.64)

a *P* value and odd ratio(OR) were adjusted by sex, age, smoking, drinking and H.pylori infection. *P* value OR and corresponding 95% interval confidence were calculated to measure the association between hypermethylation of CpG site and the risks of different groups

b the number of hypomethylated cases

c H:hypermethylation. GC: gastric cancer; AG: atrophic gastritis; CON: control; OR: odd ratio; CI: interval confidence.

### Relationship of hypermethylation of RNF180 AND RNF180 mRNA expression

To explore the possible effect of RNF180 promoter methylation on gene expression, we further evaluated the relationship of the hypermethylation and the expression of RNF180 mRNA using the Mann–Whitney *U* test. In tumor specimens, the *RNF180* mRNA levels were significantly lower than those in non-tumor specimens (*P* = 0.003). In addition, the *RNF180* mRNA levels were decreased in tend from hypermethylation to hypomethylation, although the *P* value did not reach the statistical significance (*P* = 0.075, Table 2).

**Table 2:** Relationship between RNF180 expression and gastric diseases, RNF180 hypermethylation

group		ΔCt (Mean ± SD)	Normalized 2-ΔΔCt	*P*
gastric diseases	non-tumor	4.58 ± 1.65	1.0 (0.32, 3.14)	Ref.
	tumor	5.58 ± 2.00	0.5 (0.13, 2.00)	**0.003**
methylation	hypomethylation	4.99 ± 1.90	1.0 (0.27, 3.73)	Ref.
	hypermethylation	6.46 ± 1.50	0.36 (0.13, 1.02)	0.075

### Effect of *H.pylori* infection on RNF180 PM

To explore the effect on methylation by *H.pylori* infection, we subdivided the CON, AG and GC groups by their *H.pylori* infection status, using the Mann–Whitney *U* test. AMR did not significantly differ between the *H.pylori^−^* and *H.pylori^+^* subgroups for in GC (*P* = 0.761) or AG (*P* = 0.581), but was higher in *H.pylori^+^* sub-group than *H.pylori^−^* subgroup among the controls (*P* = 0.012). A similar pattern was seen in the control group for HSC (*P* = 0.037; Table 3). In the *H.pylori^+^* sub-groups, we found 8 CpG sites with hypermethylation frequency > 25% of all 513 individuals. The results also showed that 3 CpG sites—M3(−165) (OR: 2.74; 95% CI: 1.37–5.47), M25(−34) (OR: 2.62; 95% CI: 1.29–5.31) and M30(+5) (OR: 2.80; 95% CI: 1.38–5.71), had higher hypermethylation risk in the *H.pylori^+^* AG subgroup; whereas M27(−26) had a higher methylation risk in the *H.pylori^−^* AG subgroup. The M30(+5) site also had a higher methylation risk in the *H.pylori^+^* CON subgroup (Table 4).

**Table 3:** Relationship between *H.pylori* infection and DNA methylation of RNF180 promoter area in different gastric diseases

Group	*H.pylori*	*n*	Average Methylation Rate		Methylated CpG sites count		Hypermethylated CpG sites count	
			Median (25th, 75th)	*P*	Median (25th, 75th)	*P*	Median (25th, 75th)	*P*
GC	(−)	84	0.28 (0.15, 0.36)	0.761	14 (3.5, 23.75)	0.744	5 (0, 8)	0.697
	(+)	75	0.26 (0.17, 0.34)		14 (4, 21)		4 (0, 7)	
AG	(−)	55	0.23 (0.09, 0.31)	0.581	9 (1, 16)	0.494	4 (0, 6)	0.634
	(+)	131	0.24 (0.21, 0.29)		10 (7, 14)		4 (3, 5)	
CON	(−)	143	0.05 (0.03, 0.12)	**0.012**	0 (0, 1)	0.108	0 (0, 0)	0.037
	(+)	25	0.08 (0.51, 0.32)		0 (0, 19)		0 (0, 3)	

*P* value for Mann-Whitney *U* test comparing the difference of variables between *H.pylori* positive and negative groups. GC: gastric cancer; AG: atrophic gastritis; CON:control.

**Table 4:** Association between *H.pylori* infection and the risks of Hypermethylation frequency in different gastric diseases

*H.pylori*		GC		AG		CON	
		(−)^a^	(+)^b^	(−)	(+)	(−)	(+)
M3	*n* (%)	84 (52.8)	75 (47.2)	55 (29.6)	131 (70.4)	143 (85.1)	25 (14.9)
	Hyperc	31 (53.4)	27 (46.6)	25 (22.7)	85 (77.3)	6 (75.0)	2 (25.0)
	Hypod	53 (52.5)	48 (47.5)	30 (39.5)	46 (60.5)	137 (85.6)	23 (11.4)
	*P*		0.769		**0.004**		0.509
	CI (95%)	1 (Ref.)	0.91 (0.47, 1.76)	1 (Ref.)	**2.74 (1.37, 5.47)**	1 (Ref.)	2.33 (0.19, 28.88)
M5	Hyper	22 (56.4%)	17 (43.6%)	5 (35.7%)	9 (64.3%)	4 (80.0%)	1 (20.0%)
	Hypo	62 (51.7%)	58 (48.3%)	50 (29.1%)	122 (70.9%)	139 (85.3%)	24 (14.7%)
	*P*		0.694		0.515		0.286
	CI (95%)	1 (Ref.)	0.86 (0.41, 1.82)	1 (Ref.)	0.67 (0.20, 2.23)	1 (Ref.)	4.03 (0.31, 52.06)
M7	Hyper	23 (54.8%)	19 (45.2%)	27 (25.0%)	81 (75.0%)	7 (87.5%)	1 (12.5%)
	Hypo	61 (52.1%)	56 (47.9%)	28 (35.9%)	50 (64.1%)	136 (85.0%)	24 (15.0%)
	*P*		0.574		0.064		0.798
	CI (95%)	1 (Ref.)	0.81 (0.39, 1.69)	1 (Ref.)	1.87 (0.97, 3.62)	1 (Ref.)	0.75 (0.84, 6.69)
M8	Hyper	21 (56.8%)	16 (43.2%)	17 (29.3%)	41 (70.7%)	7 (87.5%)	1 (12.5%)
	Hypo	63 (51.6%)	59 (48.4%)	38 (29.7%)	90 (70.3%)	136 (85.0%)	24 (15.0%)
	*P*		0.393		0.967		0.827
	CI (95%)	1 (Ref.)	0.71 (0.33, 1.55)	1 (Ref.)	1.02 (0.51, 2.03)	1 (Ref.)	0.782 (0.09, 7.05)
M20	Hyper	26 (54.2%)	22 (45.8%)	18 (23.4%)	59 (76.6%)	6 (60.0%)	4 (40.0%)
	Hypo	58 (52.3%)	53 (47.7%)	37 (33.9%)	72 (66.1%)	137 (86.7%)	21 (13.3%)
	*P*		0.76		0.071		0.089
	CI (95%)	1 (Ref.)	0.90 (0.44, 1.81)	1 (Ref.)	1.88 (0.95, 3.73)	1 (Ref.)	3.34 (0.83, 13.41)
M25	Hyper	44 (54.3%)	37 (45.7%)	31 (23.5%)	101 (76.5%)	17 (73.9%)	6 (26.1%)
	Hypo	40 (51.3%)	38 (48.7%)	24 (44.4%)	30 (55.6%)	126 (86.9%)	19 (13.1%)
	*P*		0.499		**0.007**		0.125
	CI (95%)	1 (Ref.)	0.80 (0.42, 1.53)	1 (Ref.)	**2.62 (1.29, 5.31)**	1 (Ref.)	2.37 (0.79, 7.10)
M27	Hyper	23 (50.0%)	23 (50.0%)	11 (55.0%)	9 (45.0%)	7 (63.6%)	4 (36.4%)
	Hypo	61 (54.0%)	61 (54.0%)	44 (26.5%)	122 (73.5%)	136 (86.6%)	21 (13.4%)
	*P*		0.623		0.014		0.052
	CI (95%)	1 (Ref.)	1.19 (0.59, 2.41)	1 (Ref.)	0.30 (0.11, 0.78)	1 (Ref.)	4.02 (0.99, 16.29)
M30	Hyper	45 (58.4%)	32 (41.6%)	33 (24.1%)	104 (75.9%)	12 (66.7%)	6 (33.3%)
	Hypo	39 (47.6%)	43 (52.4%)	22 (44.9%)	27 (55.1%)	131 (87.3%)	19 (12.7%)
	*P*		0.133		**0.005**		**0.031**
	CI (95%)	1 (Ref.)	0.61 (0.32, 1.16)	1 (Ref.)	**2.80 (1.38, 5.71)**	1 (Ref.)	**3.62 (1.13, 11.63)**

a *H.pylori* infection positive

b *H.pylori* infection negative

c Hypermethylated cases

d Hypomethylated or no-methylation cases.

*P* value was adjusted by sex age smoking and drinking. *P* value OR and corresponding 95% interval confidence were calculated to measure the association between *H.pylori* infection and the risks of hypermethylated CpG site. GC: gastric cancer; AG: atrophic gastritis; CON: control; OR: odd ratio; CI: interval confidence.

## DISCUSSION

Products of tumor suppressor genes regulate various cellular functions, and expression of these products can be modified by DNA methylation [22]. Under methylated conditions, RNA polymerase cannot effectively combine with the promoter and thus transcribe the gene, which can lead to tumorigenesis and development of cancer [23]. *RNF180* is a recently discovered suppressor gene; its product, Rines, is a membrane-bound E3 ubiquitin ligase with a coiled-coil domain and RING finger [11]. Function studies discovered the *RNF180* product could activate apoptosis by up-regulating the factors TIMP3 and CDK2A. It also could inhibit cell proliferation by up-regulating anti-proliferation factors MTSS1 and CDKN2A [12]. Further studies showed *RNF180* PM occurred more frequently in GC tissues, and *RNF180* was thus less expressed in patients with GC [13]. *RNF180* can inhibit tumorigenesis, and its methylation is related to poor prognosis [13, 14]. However, until now, the relationship between the *RNF180* PM and the risk of GC or its precancerous condition had not been deeply analyzed. To our knowledge, this is the first report about *RNF180* PM and risk of GC or AG. We calculated differences in methylation distribution between CON, AG and GC groups, to identify CpG sites with high sensitivity in distinguishing AG or GC from normal controls. We explored the relationship of the hypermethylation and the expression of RNF180 mRNA and also analyzed the effect of *H.pylori* infection on *RNF180* PM.

First, we compared differences in AMR among the control group (CON), atrophy gastritis group (AG) and gastric cancer group (GC) and found AMR significantly differed between CON, AG and GC, increasing with gastric disease severity (GC > AG > CON). We further evaluated the diagnosis efficacy of AMR. At > 0.153, AMR distinguished GC + AG from controls at 78.6% sensitivity and 77.4% specificity (AUC = 0.802); at > 0.316, AMR distinguished GC from AG + CON was 41.5% sensitive and 87.9% specific (AUC = 0.685).

Reportedly, PM of tumor suppressor genes (*RUNX3*, *CDH1*, *CDH13*, *DAPK*, *GSTP1*, etc.) may gradually accumulate with the severity of disease and stimulation of environment factors [24, 25]. We speculate that AG may be part of gastric carcinogenesis, in which *RNF180* PM gradually increases, especially the average methylation rate of the *RNF180* promoter area. As overall methylation increases over time, RNA polymerase is less able to combine with the promoter area, with expression and translation of *RNF180* products down-regulating further, eventually silencing *RNF180* [12]. When this process hit a certain level, the cells could undergo an oncologic change [26]. Thus, *RNF180* PM is associated with the transformation of gastric cancer.

We therefore propose that AMR could be a biomarker for gastric cancer and its precursor, AG. At AMR > 0.153, we found the highest possibility that gastric tissue was no longer healthy, but was either gastric cancer or precancerous AG, with AMR > 0.316 to be the best cut-off value between GC and AG. Thus, among patients who were already suffering from AG, an *RNF180* PM average rate > 0.316 could be considered an indicator of high likelihood of gastric cancer, and of a need for intervention or treatment.

We also analyzed the distribution of methylated or hypermethylated CpG sites count (MSC or HSC) in the CON, AG and GC groups. The results showed that HSC increased gradually with disease severity (GC > AG > CON), although it did not significantly differ between the GC and AG groups. We also found that MSC > 4, among a total 31 CpG sites, was 78.6% sensitive and 77.4% specific (AUC = 0.772) in identifying GC + AG; and MSC > 15 was 81.9% specific in predicting GC. When HSC > 1 among the 31 targeted CpG sites, it was 74.5% sensitive and 83.3% specific in identifying GC and AG (AUC = 0.813); HSC > 6 was 92.1% specific in predicting GC. The results were consistent previous results with AMR. Beside all these results, Therefore, MSC and HSC could also be indicators of GC or GC + AG, and generally rise with the severity of disease. Considering MSC and HSC results were shown almost the same, we advice that these results could be used according to the actual needs. For example, if we want to use lower sensitive method such as MSP or COBRA to identification GC or AG we could use hypermethylated CpG site as a biomarker for primer designing. Besides methylated CpG site can be used to achieve higher sensitivity, so that may achieve early detection of precancerous or gastric cancer.

To find CpG sites with hypermethylation that is most indicative of AG or GC, we further selected hypermethylation occurrence frequency in gastric cancer or atrophic gastritis over than 25% among all 513 individuals are been analyzed. We found that in 5 CpG sites—M3(−165), M7 (−133), M20(−57), M25(−34) and M30(+5)—hypermethylation frequency was highest in the AG group, second-highest in the GC group, and lowest in the control group, and their differences were statistically significant. Besides M5(−148) and M27(−26) hypermethylation frequency was higher in GC group than Non-GC group (AG + CON). Hypermethylation of M5(−148), M27(−26) CpG sites were associated with 5.85 OR (95% CI: 3.20–10.69) and 4.27 OR (95% CI: 2.54–7.19) in GC risk, respectively. Reportedly, 3 CpG sites in the *DACT1* gene promoter are related to GC prognosis [27]. Another study showed that hypermethylation of certain CpG sites were related to AG severity [28]. Our study found 5 CpG sites in *RNF180* promoter region that were related to AG progression, and 2 CpG sites related to GC risk. These specific CpG sites could be used for early warning of precancerous disease or gastric cancer.

In the previous study, RNF180 transcript was identified to be specially silenced or down-regulated in gastric cancer cells and primary gastric cancer tissues, and the promoter methylation was found to directly mediate RNF180 transcription silencing *in vitro* [12]. In this study, we detected the quantitative methylation of RNF180 promoter and RNF180 mRNA expression simultaneously in gastric cancer and paired non-tumor tissues. The mRNA expressive level of RNF180 was demonstrated to be significantly lower in tumor tissues than that in non-tumor tissues, and with the same tend as which in hypermethylation tissues than that in hypomethylation tissues. The results of RNF180 gene expression were consistent with Deng's report [13], and which further suggesting that the aberrant RNF180 mRNA transcription might be resulted from the DNA promoter methylation *in vivo*.

*H.pylori* as a class I carcinogen involved in gastric carcinogenesis, and was considered to affect some of tumor suppressor genes through hypermethylation [29]. To determine whether *H.pylori* infection affected methylation of *RNF180* promoter region, we further stratified the CON, AG and GC groups by *H.pylori* infection to calculate differences of AMR, MSC and HSC between the *H.pylori^−^* and *H.pylori^+^* subgroups. We found that AMR and HSC in CON/*H.pylori^+^* subgroup were higher than in the CON/*H.pylori^−^* subgroup. In the AG group, the M3(−165), M25(−34) and M30(+5) sites had increased hypermethylation risks of 2.74 folds (95% CI: 1.37–5.47), 2.62 folds (95% CI: 1.29–5.31), 2.80 folds (95% CI: 1.38–5.71), respectively, in the *H.pylori^+^* subgroup than in the *H.pylori^−^* subgroup; whereas hypermethylation of the M27(−26) site was higher in *H.pylori^−^* subgroup than in the *H.pylori^+^* subgroup. However, only 9 cases showed M27(−26) hypermethylated in the AG subgroup. *H.pylori* can release a variety of virulent determinants, such as CagA, VacA etc., that are involved in biological changes that eventually lead to AG or GC [21, 30]. *H.pylori* infection can also lead to abnormal methylation of *FOXD3*, E-cadherin and other genes, but the methylation status of these genes can be restored after removal of the *H.pylori* infection, with increases in the products of these genes [17–19]. *H.pylori* can affect methylation in two ways: overall CpG site methylation, and methylation of certain single CpG sites [31–33]. Our results, together with those of previous reports, indicate that *H.pylori* infection can increase overall methylation in normal or slightly superficial gastritis tissues, but after AG develops, *H.pylori* can increase hypermethylation at M3(−165), M25(−34) and M30(+5) in the *RNF180* promoter region. Thus, *H.pylori* infection may vary its modification of *RNF180* gene PM under the different disease conditions.

This study had some limitations. First, all subjects were from northern China, and we did not include other geographical or ethnic populations, which may have affected our results. However, considering that 42% of gastric cancers occur in China [34], this study has important reference value. Second, our cohort was rather small, especially the *H.pylori^+^* subgroup with a hypermethylated *RNF180* promoter. Therefore, our results should be verified and expanded with a larger and more varied study group.

In summary, this study focused on relationships between methylation of *RNF180* promoter and gastric cancer or atrophic gastritis and the effect of *H.pylori* infection on *RNF180* PM. We found, for the first time, that AMR, MSC, and HSC at particular CpG sites within *RNF180* promoter region could potentially be an early indicator of gastric cancer and atrophic gastritis. The aberrant expression of *RNF180* mRNA levels in tumor might be resulted from the DNA promoter methylation. We also found that *H.pylori* infection may vary how it modifies methylation in the *RNF180* gene promoter under different disease conditions. That is, in normal or superficial gastritis *H.pylori* tend to affect overall methylation status rather than hypermethylating certain CpG sites. But in atrophic gastritis, *H.pylori* was more likely to affect certain CpG sites rather than the overall methylation status. This study provides an experimental basis to evaluate *RNF180* promoter methylation as a biomarker for early warning and diagnosis of gastric cancer or atrophic gastritis.

## MATERIALS AND METHODS

### Sample collection

This study was approved by the Human Ethics Review Committee of the First Affiliated Hospital of China Medical University (Shenyang, China). All subjects gave informed consents in accordance with the declaration of Helsinki and its latest revision. We enrolled a total of 513 subjects who had undergone endoscopic examination at the Zhuanghe Gastric Diseases Screening Program and the Surgical Oncology Department in the First Affiliated Hospital of China Medical University between July, 2008 and June, 2010. Out of 513 tissue samples, 168 were normal gastric mucosa or slightly superficial gastritis tissues for the control group (CON), 186 were AG tissues, and 159 were GC tissues. In addition, we collected 55 pairs of fresh tumor tissues and non-tumor tissues from GC patients who underwent curative gastrectomy between 10, 2012 and 10, 2015 at the First Affiliated Hospital of China Medical University for RNF180 gene expression analysis. All the gastric mucosa tissues were histologically verified and their corresponding histological information was collected. Atrophic gastritis and superficial gastritis were classified by the Sydney classification system [20]. Although AG can be histologically classified as mild, moderate or severe, only moderate or severe AG were included in this study. We diagnosed CON tissues as normal stomach, or as mild superficial gastritis if mesenchymal lymphocyte counts were < 30% after excluding other systemic and gastric diseases. None of the patients had received preoperative radiotherapy or chemotherapy. We collected subjects' age, sex and other relevant clinical information by questionnaire and computerized medical records. Their GC histological diagnoses were based on the World Health Organization's criteria [16]. Participants' clinicopathological characteristics are listed in Table 5.

**Table 5:** Clinicopathological characteristics

		GC (%)	AG (%)	CON (%)	*P* value
Sex	Male	105 (66.0)	112 (60.2)	99 (58.9)	
	Female	54 (34.0)	74 (39.8)	69 (41.1)	0.363
Age	Ave. ± Std.	58.9 ± 11.7	53.4 ± 8.3	54.0 ± 7.9	< 0.001
Smoking	(−)	87 (54.7)	133 (71.4)	119 (70.8)	
	(+)	72 (45.3)	53 (28.5)	49 (29.2)	0.001
Drinking	(−)	104 (65.4)	144 (77.4)	129 (76.8)	
	(+)	55 (34.6)	42 (22.6)	39 (23.2)	0.022
*H.pylori*	(−)	84 (52.8)	55 (29.6)	143 (85.1)	
	(+)	75 (47.2)	131 (70.4)	25 (14.9)	< 0.001
Total	513	159	186	168	

GC: gastric cancer; AG: atrophic gastritis; CON:control.

### DNA extraction and sodium bisulfite modification

We extracted genomic DNA from 20-μm sections of paraffin-embedded gastric epithelial tissues using paraffin tissue extraction kit (TianGen Biochemistry, Beijing). Sodium bisulfite modification of DNA was preceded by use of the Zymo DNA Methylation-Gold kit (Zymo Research, US). All procedures followed the manufacturers' instructions.

### Enzyme-linked immunosorbent assay (ELISA) for *H.pylori* infection

Serum immunoglobulin (Ig) G antibodies to *H. pylori* were detected by *H. pylori*–IgG enzyme-linked immunosorbent assay (ELISA; Biohit, Helsinki, Finland). Antibody titers were quantified by optical density readings according to manufacturer's protocol; titers > 34 EIU (threshold value) were considered positive for *H.pylori* infection.

### Bisulfite genomic sequencing

All samples were quantitatively analyzed using bisulfite genomic sequencing (BGS) for the *RNF180* promoter area. We amplified the *RNF180* promoter area from −224 to +94, for a 319-bp fragment, with these primers: F: 5′-GTGGTTTTGGTAAGGGGATGATCC-3′; R: 5′-AACAACCAAACTCTAAAAACTC-3′ [13]. All methylation PCRs were used HotStart Taq 2.0 Version (Takara, Japan), and these conditions: initial denaturation at 94°C for 3 min; 45 cycles (94°C for 20 sec, 58.5°C for 30 sec, 72°C for 45 sec); followed by a final extension at 72°C for 10 minutes, with a final termination at 4°C. All PCR products were electrophoresed with 2% agarose gel, and staining with Genefinder (Xiamen, Zeeshan Biotechnology); remaining PCR products that shown bright band were used for backward sequence analysis after product purification.

According to the theory of bisulfite modification, unmethylated cytosine changes into uracil, and uracil is replaced by thymine during PCR; whereas methylated cytosine remains the same. The methylation rate of forward sequencing was: Meth% = [C/(C + T)]*100%. Therefore, complementary base pairing should show a methylation rate of reverse sequencing as: Meth% = [G/(G+A)]*100%. The average methylation rate (AMR; the average methylation rate for all CpG sites in each sample) methylated (methylation rate > 0.25) CpG sites count (MSC) and hypermethylated (methylation rate > 0.50) CpG sites count (HSC) was used to quantify methylation status.

In order to further confirm credibility test result, we randomly selected 10 cases of cloning sequencing DNA samples as quality control. The purified PCR products were cloned into the pUC18-T vector (Biodee, Beijing, China), and ten clones for each sample were randomly selected and sequenced (Beijing, Genomics Biotechnology). No significant differences were found between these two methods (*P* = 0.648, Supplementary Table S2).

### Quantitative real-time PCR analysis of RNF180 mRNA expression

55 pairs of gastric cancer tissues and adjacent non-cancer tissues were detected the RNF180 expression by using the quantitative real-time PCR for demonstration the difference of RNF180 mRNA expression between two groups of tissues. RNA was extracted using TRIzol reagent (Life Technologies, Carlsbad, CA) according to the manufacturer's instructions. Total RNA was converted into complementary DNA using Quantscript RT kit (Tiangen Biotech, Beijing, China). The mRNA levels specific for *RNF180* genes and an internal-control gene glyceraldehyde 3-phosphate dehydrogenase (*GAPDH*) were examined using SYBR Premix Ex *Taq* II (TaKaRa Biotech, Dalian, China) according to the manufacturer's protocol. Primers designed and utilized for *RNF180* was as follows: Forward sequence: 5′-GTGCAGTGTGTCTGGACGTT-3′, and Reverse sequence: 5′-AATGGGCATGGAGTGCTTGA-3′. Primers designed and utilized for *GAPDH* was as follows: Forward sequence: 5′-TGCACCACCAACTGCTTAG-3′, and Reverse sequence: 5′-GGATGCAGGGATGATGTTCC-3′. Melting curve analysis was performed to exclude the presence of non-specific products and primer-dimers. Each reaction was performed in duplicates, and no-template controls were included in each experiment.

The relative quantification of gene mRNA expression was calculated using the 2^*−ΔΔCt*^ method [21]. The expression levels of *RNF180* were normalized to those of *GAPDH* in each sample using the equation: ΔCt (delta Ct) = Ct_target_−Ct_GAPDH_. Relative expression levels were derived from ΔCt values as 2^*−ΔCt*^. The relative expression of tissues with tumor or hypermethylation were set to a unity, and the relative expression of tissues with non-tumor or hypomethylation were expressed relative to those of tissues with tumor or hypermethylation, thus deriving normalized 2^*−ΔΔCt*^ values.

### Statistical analysis

All statistical analysis was performed using SPSS 20.0 software (SPSS, Chicago, IL, USA). The Paired samples *t*-test was used to compare differences of the methods between Direct sequencing and Cloning sequencing. The Mann–Whitney *U* test was used to compare differences in AMR, MSC, HSC and relative mRNA levels among different groups. Receiver operating characteristic (ROC) curves and areas under curves (AUC) were used to analyze GC, AG and CON groups with regard to AMR, MSC and HSC. Multivariate logistic regression with adjustments for age, sex, smoking, drinking and *H.pylori* infection was used to assess associations between hypermethylation frequency of individual CpG sites and disease risk, and hypermethylation risk for *H.pylori* infection.

## SUPPLEMENTARY MATERIALS TABLES



## Abbreviations

GC: Gastric cancer
AG: Atrophic gastritis
CON: Control
AMR: Methylation rates
MSC: Methylated CpG site counts
HSC: Hypermethylated CpG site counts
PM: Promoter methylation
H.pylori: Helicobacter pylori
ELISA: Enzyme-linked immunosorbent assay
ROC: Receiver operating characteristic
AUC: Curves and areas under curves
