# Supplementary material for: Promoter methylation of RNF180 is associated with H.pylori infection and serves as a marker for gastric cancer and atrophic gastritis
Source: Oncotarget. 2016 Apr 1;7(17):24800–9. doi: 10.18632/oncotarget.8523 (PMC5029743; doi:10.18632/oncotarget.8523)
Supplement: Supplementary file 1 [file oncotarget-07-24800-s001.pdf]

Promoter methylation of *RNF180* is associated with *H.pylori* infection and serves as a marker for gastric cancer and atrophic gastritis

Supplementary Materials

Supplementary Table S1: Distribution of hypermethylation frequency in each group

		Frequency					Frequency		
**Name**	**Location**	**GC (%)**	**AG (%)**	**CON (%)**	**Name**	**Location**	**GC (%)**	**AG (%)**	**CON (%)**
M1	–182	13 (8.2)	2 (1.1)	3 (1.8)	M17	–77	20 (12.6)	5 (2.7)	3 (1.8)
M2	–167	14 (8.8)	7 (3.8)	9 (1.8)	M18	–65	11 (6.9)	11 (5.9)	2 (1.2)
M3	–165	0 (0.0)	0 (0.0)	1 (0.6)	M19	–61	13 (8.2)	9 (4.9)	2 (1.2)
M4	–162	16 (10.1)	6 (3.2)	2 (1.2)	***M20***	–57	***48 (30.2)***	***77 (41.6)***	***10 (6.0)***
M5	–148	***39 (24.5)***	***14 (7.6)***	***5 (3.0)***	M21	–54	36 (22.6)	18 (9.7)	9 (5.4)
M6	–146	18 (11.3)	6 (3.2)	0 (0.0)	M22	–49	18 (11.3)	8 (4.3)	2 (1.2)
M7	–133	***42 (26.4)***	***108 (58.4)***	***8 (4.8)***	M23	–44	10 (6.3)	10 (5.4)	1 (0.6)
M8	–130	***37 (23.3)***	***58 (31.4)***	***8 (4.8)***	M24	–40	10 (6.3)	3 (1.6)	2 (1.2)
M9	–127	20 (12.6)	10 (5.4)	3 (1.8)	***M25***	–34	***81 (50.9)***	***132 (71.4)***	***23 (13.7)***
M10	–121	14 (8.8)	9 (4.9)	3 (1.8)	M26	–32	30 (18.9)	13 (7.0)	5 (3.0)
M11	–119	18 (11.3)	12 (6.5)	3 (1.8)	***M27***	–26	***46 (28.9)***	***20 (10.8)***	***11 (6.5)***
M12	–117	15 (9.4)	7 (3.8)	1 (0.6)	M28	–7	14 (8.8)	8 (4.3)	3 (1.8)
M13	–112	13 (8.2)	7 (3.8)	2 (1.2)	M29	–3	15 (9.4)	12 (6.5)	3 (1.8)
M14	–100	8 (5.0)	5 (2.7)	2 (1.2)	***M30***	+5	***77 (48.4)***	***137 (74.1)***	***18 (10.7)***
M15	–94	9 (5.7)	7 (3.8)	2 (1.2)	M31	+7	26 (16.4)	9 (4.9)	3 (1.8)
M16	–87	11 (6.9)	7 (3.8)	1 (0.6)	Total		159 (100.0)	185 (100.0)	168 (100.0)

Notes: GC: gastric cancer; AG: atrophic gastritis; CON: control.

Supplementary Table S2: Comparison of direct sequencing with cloning sequencing results

Case No	Average Methylation Rate		*P*
	direct Sequencing	cloning Sequencing	
1	0.21	0.44	0.648
3	0.25	0.26	
5	0.18	0.04	
9	0.27	0.45	
15	0.04	0.06	
25	0.76	0.65	
27	0.25	0.40	
31	0.43	0.24	
33	0.25	0.36	
43	0.45	0.41	
